# Supplementary material for: Resolving the full spectrum of human genome variation using Linked-Reads
Source: Genome Res. 2019 Apr;29(4):635–45. doi: 10.1101/gr.234443.118 (PMC6442396; doi:10.1101/gr.234443.118)
Supplement: Supplemental Material [file supp_gr.234443.118_Supplemental_longranger-2.2.zip › longranger-2.2.tar.bz2!/lr2.2-public/lib/stan/cmdstan/stan/doc/api/html/classstan_1_1math_1_1_l_d_l_t__factor_3_01_t_00_01_r_00_01_c_01_4.html]

Stan: stan::math::LDLT\_factor< T, R, C > Class Template Reference


|  |  |
| --- | --- |
|  | Stan  2.5.0  probability, sampling & optimization |


- Main Page
- Related Pages
- Namespaces
- Classes
- Files

- Class List
- Class Index
- Class Hierarchy
- Class Members

All Classes Namespaces Files Functions Variables Typedefs Enumerations Enumerator Friends Macros Pages

- stan
- math
- LDLT\_factor< T, R, C >

Public Types |
Public Member Functions |
Public Attributes |
List of all members

stan::math::LDLT\_factor< T, R, C > Class Template Reference

LDLT\_factor is a thin wrapper on Eigen::LDLT to allow for reusing factorizations and efficient autodiff of things like log determinants and solutions to linear systems.
More...

`#include <LDLT_factor.hpp>`

|  |  |
| --- | --- |
| Public Types | |
| typedef size\_t | size\_type |
|  | |
| typedef double | value\_type |
|  | |

|  |  |
| --- | --- |
| Public Member Functions | |
|  | LDLT\_factor () |
|  | |
|  | LDLT\_factor (const Eigen::Matrix< T, R, C > &A) |
|  | |
| void | compute (const Eigen::Matrix< T, R, C > &A) |
|  | |
| bool | success () const |
|  | |
| T | log\_abs\_det () const |
|  | |
| void | inverse (Eigen::Matrix< T, R, C > &invA) const |
|  | |
| template<typename Rhs > | |
| const   Eigen::internal::solve\_retval  < Eigen::LDLT< Eigen::Matrix  < T, R, C > >, Rhs > | solve (const Eigen::MatrixBase< Rhs > &b) const |
|  | |
| Eigen::Matrix< T, R, C > | solveRight (const Eigen::Matrix< T, R, C > &B) const |
|  | |
| Eigen::Matrix< T,   Eigen::Dynamic, 1 > | vectorD () const |
|  | |
| Eigen::LDLT< Eigen::Matrix< T,   R, C > > | matrixLDLT () const |
|  | |
| size\_t | rows () const |
|  | |
| size\_t | cols () const |
|  | |

|  |  |
| --- | --- |
| Public Attributes | |
| size\_t | N\_ |
|  | |
| boost::shared\_ptr< Eigen::LDLT  < Eigen::Matrix< T, R, C > > > | \_ldltP |
|  | |

## Detailed Description

### template<int R, int C, typename T> class stan::math::LDLT\_factor< T, R, C >

LDLT\_factor is a thin wrapper on Eigen::LDLT to allow for reusing factorizations and efficient autodiff of things like log determinants and solutions to linear systems.

After the constructor and/or compute() is called users of LDLT\_factor are responsible for calling success() to check whether the factorization has succeeded. Use of an LDLT\_factor object (e.g., in mdivide\_left\_ldlt) is undefined if success() is false.

It's usage pattern is:

Eigen::Matrix<T,R,C> A1, A2;

LDLT\_factor<T,R,C> ldlt\_A1(A1);

LDLT\_factor<T,R,C> ldlt\_A2;

ldlt\_A2.compute(A2);

Now, the caller should check that ldlt\_A1.success() and ldlt\_A2.success() are true or abort accordingly. Alternatively, call check\_ldlt\_factor().

Note that ldlt\_A1 and ldlt\_A2 are completely equivalent. They simply demonstrate two different ways to construct the factorization.

Now, the caller can use the LDLT\_factor objects as needed. For instance

x1 = mdivide\_left\_ldlt(ldlt\_A1,b1);

x2 = mdivide\_right\_ldlt(b2,ldlt\_A2);

d1 = log\_determinant\_ldlt(ldlt\_A1);

d2 = log\_determinant\_ldlt(ldlt\_A2);

Definition at line 56 of file LDLT\_factor.hpp.

## Member Typedef Documentation

template<int R, int C, typename T >

typedef size\_t stan::math::LDLT\_factor< T, R, C >::size\_type

Definition at line 111 of file LDLT\_factor.hpp.

template<int R, int C, typename T >

typedef double stan::math::LDLT\_factor< T, R, C >::value\_type

Definition at line 112 of file LDLT\_factor.hpp.

## Constructor & Destructor Documentation

template<int R, int C, typename T >

|  |  |  |  |  |
| --- | --- | --- | --- | --- |
| stan::math::LDLT\_factor< T, R, C >::LDLT\_factor | ( |  | ) |  |

inline

Definition at line 58 of file LDLT\_factor.hpp.

template<int R, int C, typename T >

|  |  |  |  |  |  |
| --- | --- | --- | --- | --- | --- |
| stan::math::LDLT\_factor< T, R, C >::LDLT\_factor | ( | const Eigen::Matrix< T, R, C > & | *A* | ) |  |

inline

Definition at line 61 of file LDLT\_factor.hpp.

## Member Function Documentation

template<int R, int C, typename T >

|  |  |  |  |  |
| --- | --- | --- | --- | --- |
| size\_t stan::math::LDLT\_factor< T, R, C >::cols | ( |  | ) | const |

inline

Definition at line 109 of file LDLT\_factor.hpp.

template<int R, int C, typename T >

|  |  |  |  |  |  |
| --- | --- | --- | --- | --- | --- |
| void stan::math::LDLT\_factor< T, R, C >::compute | ( | const Eigen::Matrix< T, R, C > & | *A* | ) |  |

inline

Definition at line 67 of file LDLT\_factor.hpp.

template<int R, int C, typename T >

|  |  |  |  |  |  |
| --- | --- | --- | --- | --- | --- |
| void stan::math::LDLT\_factor< T, R, C >::inverse | ( | Eigen::Matrix< T, R, C > & | *invA* | ) | const |

inline

Definition at line 85 of file LDLT\_factor.hpp.

template<int R, int C, typename T >

|  |  |  |  |  |
| --- | --- | --- | --- | --- |
| T stan::math::LDLT\_factor< T, R, C >::log\_abs\_det | ( |  | ) | const |

inline

Definition at line 81 of file LDLT\_factor.hpp.

template<int R, int C, typename T >

|  |  |  |  |  |
| --- | --- | --- | --- | --- |
| Eigen::LDLT<Eigen::Matrix<T,R,C> > stan::math::LDLT\_factor< T, R, C >::matrixLDLT | ( |  | ) | const |

inline

Definition at line 104 of file LDLT\_factor.hpp.

template<int R, int C, typename T >

|  |  |  |  |  |
| --- | --- | --- | --- | --- |
| size\_t stan::math::LDLT\_factor< T, R, C >::rows | ( |  | ) | const |

inline

Definition at line 108 of file LDLT\_factor.hpp.

template<int R, int C, typename T >

template<typename Rhs >

|  |  |  |  |  |  |
| --- | --- | --- | --- | --- | --- |
| const Eigen::internal::solve\_retval<Eigen::LDLT< Eigen::Matrix<T,R,C> >, Rhs> stan::math::LDLT\_factor< T, R, C >::solve | ( | const Eigen::MatrixBase< Rhs > & | *b* | ) | const |

inline

Definition at line 92 of file LDLT\_factor.hpp.

template<int R, int C, typename T >

|  |  |  |  |  |  |
| --- | --- | --- | --- | --- | --- |
| Eigen::Matrix<T,R,C> stan::math::LDLT\_factor< T, R, C >::solveRight | ( | const Eigen::Matrix< T, R, C > & | *B* | ) | const |

inline

Definition at line 96 of file LDLT\_factor.hpp.

template<int R, int C, typename T >

|  |  |  |  |  |
| --- | --- | --- | --- | --- |
| bool stan::math::LDLT\_factor< T, R, C >::success | ( |  | ) | const |

inline

Definition at line 73 of file LDLT\_factor.hpp.

template<int R, int C, typename T >

|  |  |  |  |  |
| --- | --- | --- | --- | --- |
| Eigen::Matrix<T,Eigen::Dynamic,1> stan::math::LDLT\_factor< T, R, C >::vectorD | ( |  | ) | const |

inline

Definition at line 100 of file LDLT\_factor.hpp.

## Member Data Documentation

template<int R, int C, typename T >

boost::shared\_ptr< Eigen::LDLT< Eigen::Matrix<T,R,C> > > stan::math::LDLT\_factor< T, R, C >::\_ldltP

Definition at line 115 of file LDLT\_factor.hpp.

template<int R, int C, typename T >

size\_t stan::math::LDLT\_factor< T, R, C >::N\_

Definition at line 114 of file LDLT\_factor.hpp.

---

The documentation for this class was generated from the following file:

- src/stan/math/matrix/LDLT\_factor.hpp


---

[ Stan Home Page ]

*© 2011–2014,
Stan Development Team.*
